# Primary Care Telehealth Initiation and Engagement Among Veterans at High Risk, 2019-2022

**DOI:** 10.1001/jamanetworkopen.2024.24921

**Published:** 2024-07-31

**Authors:** Linnaea Schuttner, Brad Mayfield, Erin Jaske, Mariah Theis, Karin Nelson, Ashok Reddy

**Affiliations:** 1 Center for Innovation and Veteran-Centered Care, Veterans Affairs Puget Sound Health Care System, Seattle, Washington; 2 Department of Medicine, University of Washington School of Medicine, Seattle

## Abstract

**Importance:**

During the COVID-19 pandemic, the Veterans Health Administration (VHA) expanded telehealth infrastructure. Understanding telehealth initiation and sustained engagement could inform future resource allocation for high-need populations.

**Objective:**

To describe and examine primary care use, including initiation, use, and engagement factors, of telehealth modalities (telephone, video visits, and secure messaging) from 2020 to 2022.

**Design, Setting, and Participants:**

This cohort study was conducted among 1 383 070 patients in the 75th or higher percentile for 90-day risk of hospitalization or mortality (using previously validated Care Assessment Need scores) engaged in VHA primary care from March 11, 2019, to March 10, 2022.

**Exposures:**

Patient sociodemographic characteristics (age, sex, race and ethnicity, and marital and housing status), health characteristics (chronic condition count, military service disability, serious mental illness, or substance use disorder diagnoses), geographic characteristics (driving distance to clinic and rural or urban location), and Federal Communications Commission–reported broadband speed among subgroups of patients at high risk categorized by telehealth use from 2020 to 2022.

**Main Outcomes and Measures:**

Primary care utilization by modality.

**Results:**

A total of 1 383 070 patients at high risk were engaged in VHA primary care in March 2020 (median age, 73.0 years [IQR, 65-80 years]; 92.4% male; 77.7% regular telehealth users in 2019). With the onset of the COVID-19 pandemic from March 2020 to March 2021, 92.7% of patients at high risk (1 158 804 of 1 250 438 retained in care) became regular telehealth users. The following year, most patients continued as telehealth users (83.4% [942 151 of 1 129 683 retained]), including 38.2% retention of users at high risk newly engaged in 2020. Between 2019 and 2022 among those living and engaged in VHA primary care, adjusted exploratory multinomial logit models estimated that new telehealth users in 2020 (both sustained or only transiently engaged) were more often Black non-Hispanic individuals with greater comorbidity burdens than those who never engaged in telehealth use (Black non-Hispanic with new persistent telehealth use: adjusted relative risk ratio [ARR], 1.18 [95% CI, 1.16-1.20]; Black non-Hispanic with transient telehealth use: ARR, 1.11 [95% CI, 1.08-1.13]; ≥5 chronic conditions with new persistent telehealth use: ARR, 1.92 [95% CI, 1.88-1.96]; ≥5 chronic conditions with transient telehealth use: ARR, 1.43 [95% CI, 1.40-1.46]).

**Conclusions and Relevance:**

This cohort study suggests that primary care telehealth initiation, use and sustained engagement differed among subgroups of patients at high risk throughout the COVID-19 pandemic. Those never or only transiently engaged with telehealth had lower illness burdens and were less likely to identify as members of racial or ethnic minority groups. Variation in telehealth use among subgroups of patients at high risk during this period could inform future resource allocation.

## Key Points

**Question:**

How did patients at high risk (≥75th percentile for 90-day risk of hospitalization) engage with primary care telehealth modalities (telephone, video visits, or secure messaging) within the Veterans Health Administration between 2019 and 2022?

**Findings:**

In this cohort study including 1 383 070 patients at high risk, among those newly engaged in telehealth with the onset of the COVID-19 pandemic, 38% remained regular telehealth users the following year. Patients had distinct patterns of primary care telehealth use during and after the COVID-19 pandemic.

**Meaning:**

This study suggests that access barriers may limit initial telehealth engagement among some patients at high risk of hospitalization or mortality, although factors associated with uptake and sustainment vary; these patterns can inform future resource allocation.

## Teaser

This cohort study examines the use of primary care telehealth services (telephone, video visits, or secure messaging) among Veterans Health Administration patients at high risk of hospitalization from 2019 to 2022.

## Introduction

During the COVID-19 pandemic, the Veterans Health Administration (VHA) rapidly expanded telehealth services, including secure messaging and video and telephone visits.^1,2^ Telehealth outreach, such as development of a VHA loaned tablet program, particularly targeted veterans at high risk of adverse events given known access barriers.^2^ However, evaluations of telehealth use among patients at high risk have been limited to early or prepandemic findings.^3,4^ After the pandemic, telehealth infrastructure has gained permanence, with nearly 50% of visits in primary care being done via video or telephone modalities.^5,6^ Understanding primary care use and telehealth engagement among high-risk patients after these changes in access could inform resource allocation and policy to optimize primary care staffing, care delivery, and alignment with patient preferences among a high-need, high-cost population.^7,8^

## Methods

### Study Design

This cohort study examined primary care use by modality among patients in the VHA at high risk of adverse hospitalization or mortality during and after the COVID-19 pandemic from March 11, 2019, to March 10, 2022. We describe primary care utilization and changes by year among high-risk patients for 3 time periods (before the pandemic [March 11, 2019, to March 10, 2020], pandemic year 1 [March 11, 2020, to March 10, 2021], and pandemic year 2 [March 11, 2021, to March 10, 2022]). Among the patients living and active in VHA primary care during all 3 years, we explored factors associated with telehealth initiation and sustained engagement among demographic and geographic subgroups of high-risk patients. This study was designated nonresearch quality improvement by the VHA Office of Primary Care in accordance with the national VHA Office of Research and Development policy^9^ and not subject to institutional review board review or exemption. This study followed the Strengthening the Reporting of Observational Studies in Epidemiology (STROBE) reporting guideline.^10^

### Data Sources

Patient and clinic characteristics were from the Veterans Affairs (VA) Corporate Data Warehouse (CDW), except as described.^11^ Primary care empanelment was determined using the Primary Care Management Module for active assignment in the period of interest. Patient death data in the CDW are updated daily and aggregate data from VHA facilities, death certificates, and National Cemetery Administration records. Race and ethnicity data were included given the potential for inequitable access to telehealth modalities by race and ethnicity.^12,13^ These data use a previously developed algorithm that aggregates values across multiple VA medical record and demographic datasets, prioritizing sources of patient self-report; categories of “Other” race are included from historic response options in some datasets and include “declined,” “multiple,” or “unknown.”^14^ Serious mental illness is an indicator based on diagnoses codes and functional assessments, established by the VA Program Evaluation Resource Center, and includes schizophrenia, schizoaffective disorder, bipolar disorder, major depressive disorder with psychosis, or posttraumatic stress disorder in a patient receiving antipsychotic medications.^15^ Veterans Affairs Priority Groups are categories of military service–connected disability or income. Chronic condition counts include 28 health conditions listed within the Gagne comorbidity index,^16^ counted if present by *International Statistical Classification of Diseases and Related Health Problems, Tenth Revision* codes within the electronic health record within 5 years prior.

Internet speed was linked by patient zip code according to the 2010 US Census Bureau census block shapefile, which is linked to availability within Federal Communications Commission Fixed Broadband Data.^17^ For our baseline covariates, these data were updated through June 2020 (as released every 6 months). Speed adequacy cut points have been previously categorized as inadequate (download speed, <25 MB/s; upload speed, <3 MB/s), adequate (download speed, ≥25 and <100 MB/s; upload speed, ≥5 and <100 MB/s), and optimal (download and upload speeds, ≥100 MB/s).^18^ Geographic location (rurality) was from the VA Planning Systems Support Group (PSSG) geocoded data, linked to patient zip codes from the US Census Bureau. Driving distance is also from PSSG data, using patient travel time to the nearest VHA primary care clinic with distances from the VA Site Tracking System. Distances are categorized as less than 64.4 km or more, consistent with definitions for care referral parameters.^19^

Veterans Health Administration Primary Care staffing ratios were included as covariates indicating clinic-level measures of support staff per primary care clinicians, obtained from the Patient Aligned Care Team Compass module within the CDW. Clinics were categorized as community affiliated or hospital affiliated per the VA Site Tracking System. Care modalities and type were defined by VA Managerial Cost Accounting procedural and encounter stop codes (eTable 1 in Supplement 1).

### Patient Population

We included veterans with an estimated risk of hospitalization or mortality in the 75th percentile or greater within 90 days (ie, high risk for this study) enrolled in VHA primary care before the start of the COVID-19 pandemic (March 10, 2020).^20^ Estimated risk refers to a percentile of probabilities using the previously validated Care Assessment Need score, a VA-specific model based on patient clinical and demographic characteristics that estimates the probability of future hospitalization or mortality in a period of time.^21,22^

### Statistical Analysis

We examined 3 time periods for descriptive trends: before the pandemic (March 11, 2019, to March 10, 2020), pandemic year 1 (March 11, 2020, to March 10, 2021), and pandemic year 2 (March 11, 2021, to March 10, 2022). For each year, we created a binary indicator of regular telehealth use for primary care encounters, defined as 1 or more video visit or secure message or greater than median per-year proportion of telephone visits to other primary care modalities. We examined use of general primary care (including women’s health) vs specialized primary care (home-based primary care, geriatrics, homeless care) for descriptive trends.

We classified cohort patients still living and active in primary care at the end of year 2 into 5 subgroups according to retroactive telehealth use as a binary factor per year across the 3-year period: never users (no telehealth use in any year); transient users (telehealth use in pandemic year 1 only); new persistent users (telehealth use in pandemic years 1 and 2); consistent users (telehealth use in all 3 years); and remaining as all others. Variation in patient-level characteristics among these subgroups for 3-year patterns of telehealth use were examined using exploratory multinomial logistic regression models with clinic-level random effects to control for differences in infrastructure and facility-level telehealth ability. Except as described above, covariates for models were from the most recent quarter in the prepandemic year. Models were also adjusted for clinic staffing ratios, community- or hospital-level affiliation, and baseline patient primary care visit use. Outcomes are presented as adjusted relative risk ratios (ARRs). Never users were set as the reference group. Patients missing covariates were dropped from the models (2201 of 1 129 683 [0.2%]) for continuous variables, while missing categorical variables were included as separate levels or aggregated into other levels for small cell counts, as follows. Patient race and ethnicity levels of Asian (6081 of 1 127 482 [0.5%]); Alaska Native, American Indian, Native Hawaiian, and Other Pacific Islander (15 109 of 1 127 482 [1.3%]); and multiracial, unknown, or other (39 036 of 1 127 482 [3.5%]) were combined. Chronic condition count of unknown (11 064 of 1 127 482 [1.0%]) was combined with condition count less than 2 (referent). Highly rural, islander, or unknown (42 914 of 1 127 482 [3.8%]) were combined with rural geography.

Dataset preparation and descriptive statistics used SAS EG, version 7.1 (SAS Institute Inc).^23^ Statistical modeling was conducted using R, version 4.3.1 (R Project for Statistical Computing),^24^ with extension mclogit or mblogit version 0.9.6.^25^ For all analyses, significance was determined using a 2-tailed type I error rate of *P* < .05.

## Results

Before the pandemic, of 5 628 379 patients enrolled in VHA primary care at 1068 facilities, 1 383 070 were at high risk. High-risk patients before the pandemic were older (median age, 73.0 years [IQR, 65-80 years] vs 67.0 years [IQR, 51-76 years]; ≥85 y, 18.5% vs 9.4%), with more men (92.4% vs 90.3%) than lower-risk patients. Among the high-risk patients before the pandemic, there were 7.0% Hispanic individuals; 0.6% non-Hispanic Asian individuals; 20.5% non-Hispanic Black individuals; 1.4% non-Hispanic Alaska Native, American Indian, Native Hawaiian, and Other Pacific Islander individuals; and 70.6% non-Hispanic White individuals. Among the lower-risk patients before the pandemic, there were 8.3% Hispanic individuals; 1.4% non-Hispanic Asian individuals; 17.6% non-Hispanic Black individuals; 1.5% non-Hispanic Alaska Native, American Indian, Native Hawaiian, and Other Pacific Islander individuals; and 68.1% non-Hispanic White individuals. Of the 1 383 070 high-risk patients before the pandemic, 1 250 438 were still living and active in primary care in pandemic year 1, and 1 129 683 were still living and active in primary care in year 2. Between the prepandemic period and year 2, 246 475 patients (97.3%) had died and 6912 (2.7%) were no longer empaneled at any site in VHA primary care. By year 2, surviving care-engaged high-risk patients were younger compared with high-risk patients before the pandemic (median age, 72.0 years [IQR, 63-78 years]), with fewer men (91.3%) and non-Hispanic White patients (69.0%), and with greater illness (high disability status, 43.9% vs 37.4%) and less social risk burden (serious mental illness, 4.0% vs 5.6%; substance use disorder, 10.0% vs 14.1%; homelessness, 1.1% vs 2.9%) (eTable 2 in Supplement 1). Across all years, most high-risk patients were cared for in general primary care teams vs specialized primary care teams (mean [SD] general primary care use by year, 94.9% [0.4%]).

**Figure 1.**
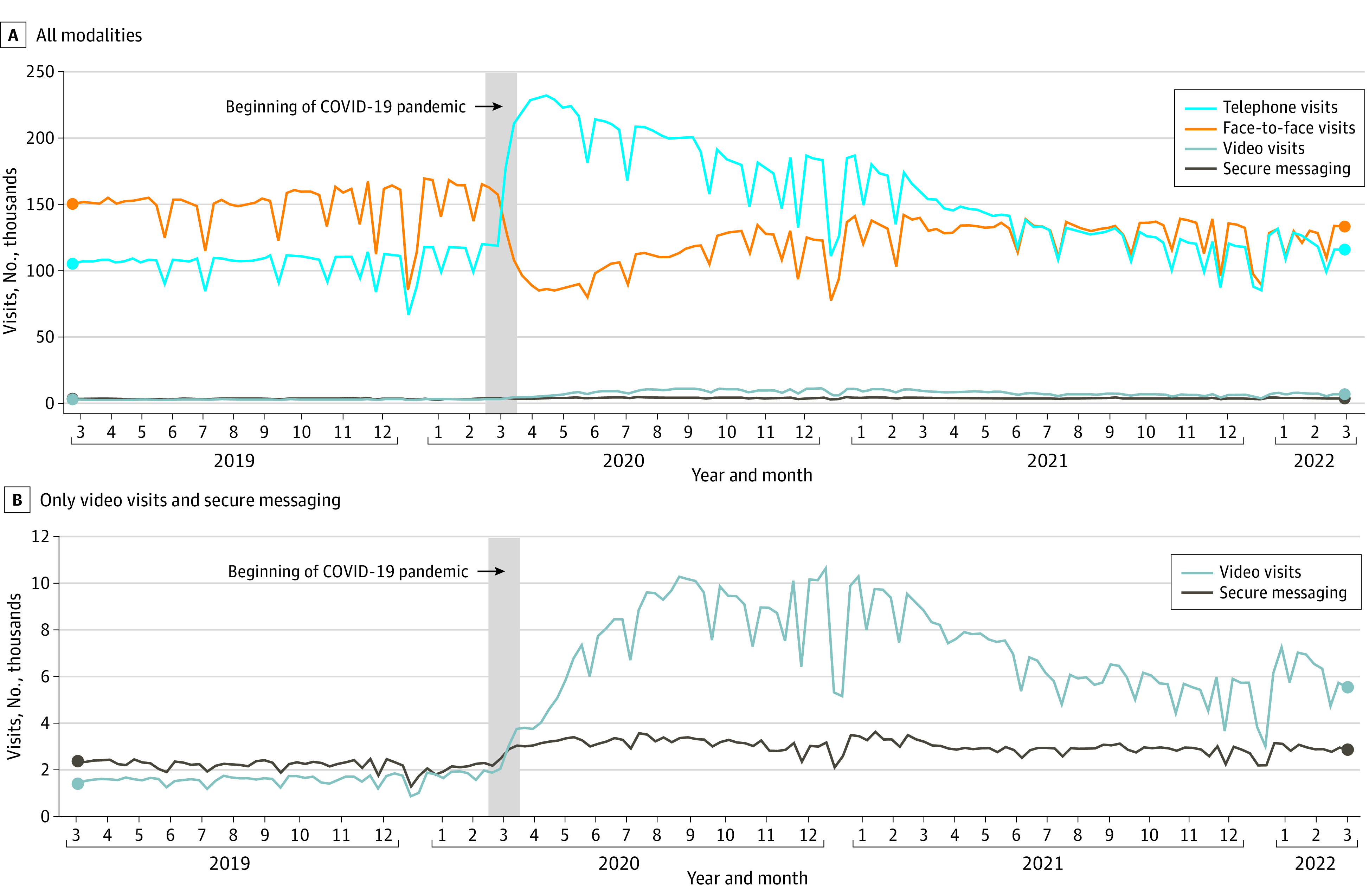
Primary Care by Visit Modality Among Veterans at High Risk of Adverse Events

Before the pandemic, 77.7% of high-risk patients (n = 1 075 217) had used telehealth (all types) and only 5.0% (n = 68 615) had also used video or secure messaging. During the pandemic, telehealth use shifted dramatically (Figure 1). In year 1, 92.7% of high-risk patients (n = 1 158 804) became regular telehealth users (all types), and 21.1% (n = 263 830) specifically used video or secure messaging. In year 2, 83.4% of high-risk patients (n = 942 151) were regular telehealth users (all types), a 38.2% retention of new telehealth users engaged from year 1. For video and secure messaging, 18.0% of high-risk patients (n = 203 074) were users during year 2, an 81.7% retention of those newly engaged with video and secure messaging during year 1.

**Figure 2.**
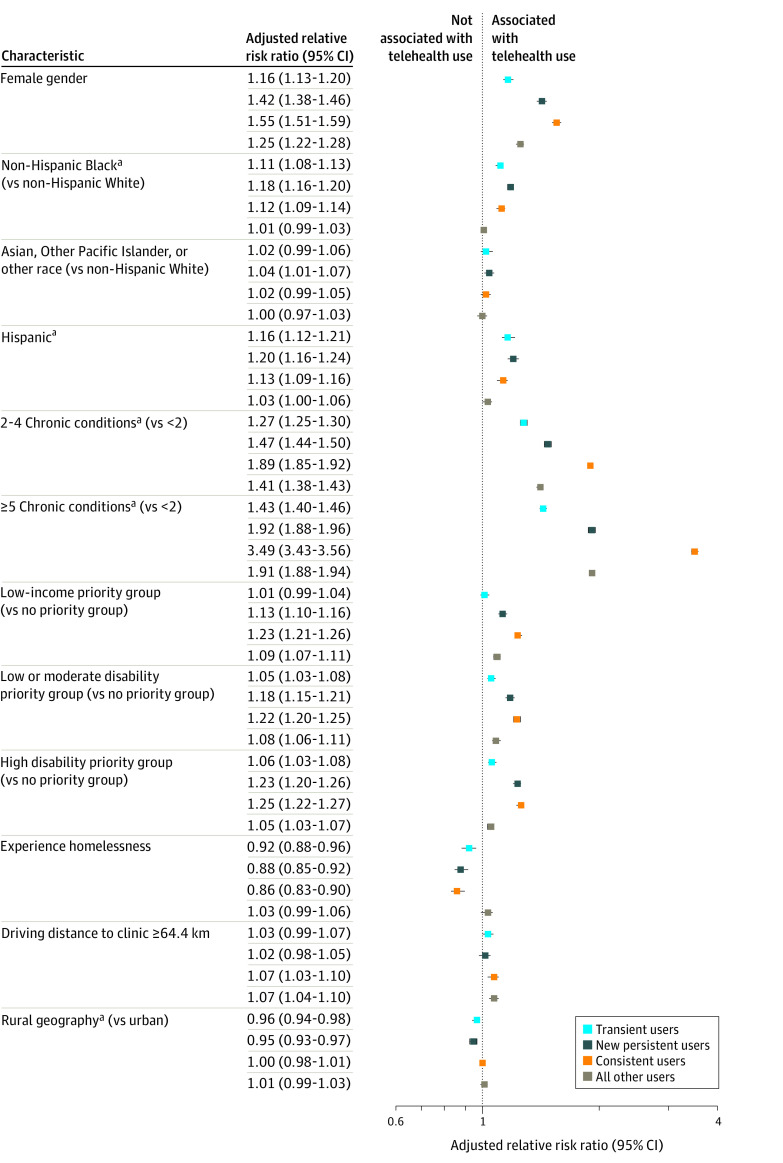
Telehealth Use Among Veterans at High Risk Engaged in Primary Care Throughout 2019 to 2022 Compared With Never Users Patients missing continuous covariates were excluded from models (n = 2201 [0.2%]). Models also adjusted for primary care staffing ratio and community vs hospital affiliation. ^a^Combined levels due to small cell sizes.

High-risk patients still living and engaged in primary care by pandemic year 2 were divided into 5 different telehealth use profiles (baseline, prepandemic characteristics in the Table). After adjustment for clinic and patient-level covariates, between 2019 and 2022 among those living and engaged in VHA primary care, adjusted exploratory multinomial logit models estimated that new telehealth users in 2020 (both sustained or only transiently engaged) were more often Black non-Hispanic individuals with greater comorbidity burdens than those who never engaged in telehealth use (Black individuals with new persistent telehealth use: ARR, 1.18 [95% CI, 1.16-1.20]; Black individuals with transient telehealth use: ARR, 1.11 [95% CI, 1.08-1.13]; ≥5 chronic conditions with new persistent telehealth use: ARR, 1.92 [95% CI, 1.88-1.96]; ≥5 chronic conditions with transient telehealth use: ARR, 1.43 [95% CI, 1.40-1.46]) (Figure 2). Those who were never users were the most likely to be men with the fewest chronic conditions and lowest disability burden at baseline. Consistent users were more likely to be women and had the highest comorbidity and disability burden. Both transient users and new persistent users were more often located in urban settings. New persistent users were proportionately those more often identifying as Black and Hispanic, women, low-income individuals, and those who had greater comorbidity burdens than transient users. Transient users had fewer chronic conditions and lower disability burdens than all other subgroups except never users. Age, marital status, serious mental illness, substance use, and internet adequacy at baseline were less characterizing of differences in ongoing telehealth use between subgroups (eFigure in Supplement 1).

**Table.** Baseline Characteristics of Veterans at High Risk Engaged in VHA Primary Care From 2019 to 2022, Grouped by Telehealth User Profile

Characteristic	Veterans, No. (%)						
	All VHA patients (N = 5 628 379)	All patients at high risk (n = 1 129 683)	Never users (n = 169 508)	Transient users (n = 120 490)	New persistent users (n = 164 442)	Consistent users (n = 302 083)	All other users (n = 373 129)
Age, median (IQR), y	68.0 (55-77)	72.0 (63-78)	73.0 (65-80)	72.0 (63-78)	72.0 (63-78)	72.0 (64-78)	72.0 (64-78)
≥85 y	537 479 (9.6)	153 003 (13.5)	30 464 (18.0)	17 188 (14.3)	20 909 (12.7)	33 296 (11.0)	51 120 (13.7)
Sex^a^							
Male	5 020 325 (90.6)	1 031 578 (91.3)	158 279 (93.4)	110 628 (91.8)	147 590 (89.8)	272 182 (90.2)	342 446 (91.8)
Female	513 549 (9.2)	97 990 (8.7)	11 153 (6.6)	9818 (8.1)	16 787 (10.2)	29 728 (9.8)	30 499 (8.2)
Race and ethnicity^b^							
Alaska Native, American Indian, Native Hawaiian, or Other Pacific Islander, non-Hispanic	77 455 (1.4)	15 148 (1.3)	2178 (1.3)	1499 (1.2)	2212 (1.4)	4243 (1.4)	5016 (1.3)
Asian, non-Hispanic	65 323 (1.2)	6206 (0.6)	798 (0.5)	625 (0.5)	996 (0.6)	1751 (0.6)	2036 (0.6)
Black, non-Hispanic	988 041 (17.5)	236 883 (21.0)	31 412 (18.5)	27 352 (22.7)	40 875 (24.9)	64 435 (21.3)	72 799 (19.5)
Hispanic	430 489 (7.6)	79 897 (7.1)	9507 (5.6)	10 140 (8.4)	16 968 (10.3)	20 833 (6.9)	22 445 (6.0)
White, non-Hispanic	3 834 741 (67.8)	752 332 (66.6)	119 822 (70.7)	76 742 (63.7)	97 729 (59.4)	200 295 (66.3)	257 731 (69.1)
Married	3 121 196 (55.5)	512 027 (45.3)	75 667 (44.7)	53 843 (44.7)	73 639 (44.8)	141 049 (46.7)	167 591 (44.9)
Chronic conditions, mean (SD), No.	3.1 (2.0)	4.6 (2.1)	4.2 (2.0)	4.4 (2.0)	4.6 (2.1)	5.1 (2.2)	4.5 (2.1)
Serious mental illness	129 865 (2.3)	69 550 (6.2)	9359 (5.5)	7583 (6.3)	11 323 (6.9)	19 191 (6.4)	21 978 (5.9)
Substance use disorder	354 011 (6.3)	171 783 (15.2)	23 348 (13.8)	19 057 (15.8)	25 611 (15.6)	45 869 (15.2)	57 694 (15.5)
High disability^c^	2 009 914 (35.8)	437 112 (38.7)	61 332 (36.2)	46 702 (38.8)	67 775 (41.2)	122 130 (40.4)	139 162 (37.3)
Hospitalized in prior year^d^	46 022 (0.8)	30 445 (2.7)	2618 (1.6)	2157 (1.8)	3201 (2.0)	11 327 (3.8)	11 135 (3.0)
Homelessness	61 528 (1.1)	35 826 (3.2)	4961 (2.9)	3867 (3.2)	5431 (3.3)	8947 (3.0)	12 541 (3.4)
Driving distance to primary care, mean (SD), km	25.6 (24.3)	24.1 (23.5)	24.6 (23.5)	23.3 (21.9)	22.2 (20.9)	24.5 (24.1)	24.9 (24.6)
Geography							
Urban	3 659 479 (65.0)	759 238 (67.2)	108 161 (63.8)	84 167 (69.9)	120 647 (73.4)	202 017 (66.9)	243 809 (65.4)
Rural	1 724 051 (30.6)	326 803 (28.9)	53 437 (31.5)	32 136 (27.7)	39 137 (23.8)	88 569 (29.3)	113 346 (30.4)
Highly rural or islands^e^	244 849 (4.8)	42 739 (3.9)	7844 (4.6)	4138 (3.4)	4557 (2.8)	11 294 (3.7)	15 782 (4.2)
CBOC	3 504 379 (62.4)	583 168 (51.6)	89 174 (52.6)	61 644 (51.2)	79 418 (48.3)	158 324 (52.4)	194 358 (52.1)
Internet speed							
Optimal	2 143 287 (38.1)	427 724 (37.9)	60 611 (35.9)	45 709 (38.0)	64 846 (39.5)	117 314 (38.9)	139 232 (37.4)
Adequate	2 008 956 (53.5)	618 593 (54.8)	95 187 (56.3)	66 406 (55.2)	89 364 (54.4)	162 916 (54.0)	204 703 (55.0)
Inadequate	412 411 (7.3)	81 898 (7.3)	13 227 (7.8)	8239 (6.7)	10 084 (6.1)	21 604 (7.2)	28 744 (7.7)

Abbreviations: CBOC, community-based outpatient clinic; VHA, Veterans Health Administration.

^a^ May not sum to 100%; sex missing not shown.

^b^ Other and unknown races and ethnicities are not shown; this category includes multiple races or response declined.

^c^ Veterans Affairs priority group is a military service–related disability or income determination.

^d^ All-cause acute hospitalizations (≥1) in 12 months prior.

^e^ Includes unknown geography.

## Discussion

Patterns of telehealth use in primary care for high-risk patients changed throughout the COVID-19 pandemic. Our study has 3 main takeaways relevant to health systems. First, we found that while telehealth primary care use increased dramatically with pandemic onset, telehealth use declined subsequently as use of in-person care rebounded. Despite growing interest in virtual care, this finding underscores the continued need for resources to support in-person care among high-risk populations.^26^

Second, we found that general primary care, as opposed to more specialized primary care teams, remained the front line for high-risk patients during and after the pandemic. Despite expected attrition due to mortality and disengagement,^27,28,29^ high-risk patients who remained in care throughout the pandemic remained at high complexity with a higher disability burden despite a slight lessening of psychosocial factors. Change in military service–connected disability is not insignificant for complexity; veterans with a high disability rating have mortality rates 2.5 times those with lower levels.^30^ This finding highlights the ongoing importance of general primary care after the pandemic for high-complexity, high-need populations, consistent with prepandemic literature.^8^

Third, several key patterns of telehealth use emerged for high-risk patients and could be applied to aid system-level resource allocation among primary care clinics. Overall, nearly 4 in 10 high-risk new telehealth users (all telehealth modalities) who initially engaged in telehealth in the early pandemic remained telehealth users during year 2. In contrast, 8 in 10 new users who initially engaged in video and secure messaging during year 1 remained users during year 2. This finding supports that telehealth initiation and adoption barriers may have been rate-limiting for many high-risk patients before the pandemic, consistent with earlier pandemic studies in broader VHA and non-VHA populations.^1,3,4^ Our work helps further clarify engagement and sustainment factors among this high-risk cohort. For example, urban clinics serving a higher proportion of patients with characteristics consistent with new persistent users might invest in programs focused on telehealth initiation barriers, such as digital tablet loans, to reduce disparities in access. In comparison, rural clinics with higher proportions of patients with characteristics consistent with never users might anticipate that staffing and resource allocation should go toward ongoing support of in-person visits.

### Limitations

This study has some limitations, including caution when generalizing beyond veteran populations and potential misclassification due to administrative data. Results are descriptive of care use among high-risk patients who remained active and alive during the period of study and are not indicative of outcomes for all patients.

## Conclusions

We examined factors associated with telehealth use for high-risk patients sustaining primary care in the VHA from 2019 to 2022. Among these patients, telehealth uptake and sustained engagement differed. We found that high-risk patients with barriers to initial access that, once addressed, led to sustained engagement were more commonly urban and those identifying as members of racial and ethnic minority groups. Patients who never engaged or only transiently engaged in telehealth before returning to in-person visits for primary care had lower illness and disability burdens. These patterns of telehealth use in primary care have implications for health systems seeking to optimize primary care staffing and clinic infrastructure, such as allocation of clinician time and resources to support access for in-person or virtual visits among their high-need, high-risk patients.

## Notes

Supplement 1. **eTable 1.** Stop Code Classifications **eTable 2.** Characteristics of High-Risk Veterans That Remained Engaged in VA Primary Care From 2019-2022 **eFigure.** Telehealth Use Among High-Risk Veterans Engaged in Primary Care Throughout 2019-2022, Adjusted Relative Risk Ratios (95% CI) Compared to Never Users (All Modelled Results)

Supplement 2.
Data Sharing Statement

## References

[1] Ferguson JM, Jacobs J, Yefimova M, Greene L, Heyworth L, Zulman DM. Virtual care expansion in the Veterans Health Administration during the COVID-19 pandemic: clinical services and patient characteristics associated with utilization. J Am Med Inform Assoc. 2021;28(3):453-462. doi:10.1093/jamia/ocaa284 33125032 PMC7665538

[2] Heyworth L, Kirsh S, Zulman D, Ferguson JM, Kizer KW. Expanding access through virtual care: the VA’s early experience with COVID-19. *NEJM Catal*. July 1, 2020. Accessed January 8, 2024. https://catalyst.nejm.org/doi/pdf/10.1056/CAT.20.0327

[3] Babaei N, Zamanzadeh V, Valizadeh L, . A scoping review of virtual care in the health system: infrastructures, barriers, and facilitators. Home Health Care Serv Q. 2023;42(2):69-97. doi:10.1080/01621424.2023.2166888 36635987

[4] Ferguson JM, Wray CM, Jacobs J, . Variation in initial and continued use of primary, mental health, and specialty video care among veterans. Health Serv Res. 2023;58(2):402-414. doi:10.1111/1475-6773.14098 36345235 PMC10012228

[5] Millman A, Huang J, Graetz I, . Patient-reported primary care video and telephone telemedicine preference shifts during the COVID-19 pandemic. Med Care. 2023;61(11):772-778. doi:10.1097/MLR.0000000000001916 37733433 PMC10592113

[6] Reed M, Huang J, Somers M, . Telemedicine versus in-person primary care: treatment and follow-up visits. Ann Intern Med. 2023;176(10):1349-1357. doi:10.7326/M23-1335 37844311 PMC11382601

[7] Zulman DM, Pal Chee C, Wagner TH, . Multimorbidity and healthcare utilisation among high-cost patients in the US Veterans Affairs Health Care System. BMJ Open. 2015;5(4):e007771. doi:10.1136/bmjopen-2015-007771 25882486 PMC4401870

[8] Chang ET, Zulman DM, Nelson KM, . Use of general primary care, specialized primary care, and other Veterans Affairs services among high-risk veterans. JAMA Netw Open. 2020;3(6):e208120. doi:10.1001/jamanetworkopen.2020.8120 32597993 PMC7324956

[9] Office of Research & Development, Department of Veterans Affairs. *Program Guide: 1200.21. VHA Operations Activities That May Constitute Research*. US Dept of Veterans Affairs; 2019. Accessed January 8, 2024. https://www.research.va.gov/resources/policies/ProgramGuide-1200-21-VHA-Operations-Activities.pdf

[10] von Elm E, Altman DG, Egger M, Pocock SJ, Gøtzsche PC, Vandenbroucke JP; STROBE Initiative. The Strengthening the Reporting of Observational Studies in Epidemiology (STROBE) statement: guidelines for reporting observational studies. Ann Intern Med. 2007;147(8):573-577. doi:10.7326/0003-4819-147-8-200710160-00010 17938396

[11] Price LE, Shea K, Gephart S. The Veterans Affairs’s Corporate Data Warehouse: uses and implications for nursing research and practice. Nurs Adm Q. 2015;39(4):311-318. doi:10.1097/NAQ.0000000000000118 26340242 PMC10071958

[12] Rivera V, Aldridge MD, Ornstein K, Moody KA, Chun A. Racial and socioeconomic disparities in access to telehealth. J Am Geriatr Soc. 2021;69(1):44-45. doi:10.1111/jgs.16904 33075143 PMC8726710

[13] Eberly LA, Kallan MJ, Julien HM, . Patient characteristics associated with telemedicine access for primary and specialty ambulatory care during the COVID-19 pandemic. JAMA Netw Open. 2020;3(12):e2031640. doi:10.1001/jamanetworkopen.2020.31640 33372974 PMC7772717

[14] Hernandez SE, Sylling PW, Mor MK, . Developing an algorithm for combining race and ethnicity data sources in the Veterans Health Administration. Mil Med. 2020;185(3-4):e495-e500. doi:10.1093/milmed/usz322 31603222

[15] Committee to Evaluate the Department of Veterans Affairs Mental Health Services, Board on Health Care Services, Health and Medicine Division, National Academies of Sciences, Engineering, and Medicine. *Evaluation of the Department of Veterans Affairs Mental Health Services*. National Academies Press; 2018:24915. 29738208

[16] Gagne JJ, Glynn RJ, Avorn J, Levin R, Schneeweiss S. A combined comorbidity score predicted mortality in elderly patients better than existing scores. J Clin Epidemiol. 2011;64(7):749-759. doi:10.1016/j.jclinepi.2010.10.004 21208778 PMC3100405

[17] Economics and Analytics. Fixed broadband deployment data from FCC form 477. Federal Communications Commission. December 29, 2022. Accessed November 17, 2023. https://www.fcc.gov/general/broadband-deployment-data-fcc-form-477

[18] O’Shea AMJ, Baum A, Haraldsson B, . Association of adequacy of broadband internet service with access to primary care in the Veterans Health Administration before and during the COVID-19 pandemic. JAMA Netw Open. 2022;5(10):e2236524. doi:10.1001/jamanetworkopen.2022.36524 36251295 PMC9577674

[19] VA MISSION Act of 2018, S.2372, 115th Cong (2017-2018). Pub L No. 115-182. Accessed January 8, 2024. https://www.congress.gov/bill/115th-congress/senate-bill/2372/text

[20] WHO Director-General’s opening remarks at the media briefing on COVID-19 - 11 March 2020. World Health Organization. March 11, 2020. Accessed August 10, 2023. https://www.who.int/director-general/speeches/detail/who-director-general-s-opening-remarks-at-the-media-briefing-on-covid-19---11-march-2020

[21] Nelson KM, Chang ET, Zulman DM, Rubenstein LV, Kirkland FD, Fihn SD. Using predictive analytics to guide patient care and research in a national health system. J Gen Intern Med. 2019;34(8):1379-1380. doi:10.1007/s11606-019-04961-4 31011959 PMC6667597

[22] Fihn SD, Francis J, Clancy C, . Insights from advanced analytics at the Veterans Health Administration. Health Aff (Millwood). 2014;33(7):1203-1211. doi:10.1377/hlthaff.2014.0054 25006147

[23] SAS. SAS Institute Inc. September 2017. Accessed January 8, 2024. https://www.sas.com/en_us/home.html

[24] R: a language and environment for statistical computing. The R Project for Statistical Computing. Accessed June 24, 2021. http://www.R-project.org

[25] Elff M. mclogit: Multinomial logit models, with or without random effects or overdispersion. Accessed April 3, 2024. http://melff.github.io/mclogit/

[26] Wong ES, Rosland AM, Fihn SD, Nelson KM. Patient-centered medical home implementation in the Veterans Health Administration and primary care use: differences by patient comorbidity burden. J Gen Intern Med. 2016;31(12):1467-1474. doi:10.1007/s11606-016-3833-9 27503440 PMC5130955

[27] Wong SYS, Zhang D, Sit RWS, . Impact of COVID-19 on loneliness, mental health, and health service utilisation: a prospective cohort study of older adults with multimorbidity in primary care. Br J Gen Pract. 2020;70(700):e817-e824. doi:10.3399/bjgp20X713021 32988955 PMC7523921

[28] Yonemoto N, Kawashima Y. Help-seeking behaviors for mental health problems during the COVID-19 pandemic: a systematic review. J Affect Disord. 2023;323:85-100. doi:10.1016/j.jad.2022.11.043 36435398 PMC9684094

[29] Ioannou GN, Locke E, Green P, . Risk factors for hospitalization, mechanical ventilation, or death among 10 131 US veterans with SARS-CoV-2 infection. JAMA Netw Open. 2020;3(9):e2022310. doi:10.1001/jamanetworkopen.2020.22310 32965502 PMC7512055

[30] Maynard C, Nelson K, Fihn SD. Disability rating and 1-year mortality among veterans with service-connected health conditions. Public Health Rep. 2018;133(6):692-699. doi:10.1177/0033354918794929 30223760 PMC6225874

