# Supplementary material for: Primary Care Telehealth Initiation and Engagement Among Veterans at High Risk, 2019-2022
Source: JAMA Netw Open. 2024 Jul 31;7(7):e2424921. doi: 10.1001/jamanetworkopen.2024.24921 (PMC11292453; doi:10.1001/jamanetworkopen.2024.24921)
Supplement: Supplement 1. — eTable 1. Stop Code Classifications eTable 2. Characteristics of High-Risk Veterans That Remained Engaged in VA Primary Care From 2019-2022 eFigure. Telehealth Use Among High-Risk Veterans Engaged in Primary Care Throughout 2019-2022, Adjusted Relative Risk Ratios (95% CI) Compared to Never Users (All Modelled Results) [file jamanetwopen-e2424921-s001.pdf]

Supplementary Online Content

Schuttner L, Mayfield B, Jaske E, Theis M, Nelson K, Reddy A. Primary care telehealth initiation and engagement among veterans at high risk, 2019-2022. *JAMA Netw Open.* 2024;7(7):e2424921.
doi:10.1001/jamanetworkopen.2024.24921

eTable 1. Stop Code Classifications
eTable 2. Characteristics of High-Risk Veterans That Remained Engaged in VA Primary Care From 2019-2022
eFigure. Telehealth Use Among High-Risk Veterans Engaged in Primary Care Throughout 2019-2022, Adjusted Relative Risk Ratios (95% CI) Compared to Never Users (All Modelled Results)

This supplementary material has been provided by the authors to give readers additional information about their work.

© 2024 Schuttner L et al. *JAMA Network Open.*

eTable 1. Stop Code Classifications

Specialized versus generalized primary care	
Specialized primary care (Home based primary care, Geriatrics, Geriatric consultations)	170, 172, 350
General primary care (General internal medicine, Women's Health, Ambulatory/primary care)	322, 323, 348

Primary care visit modality

Visit Type	Primary Stop Code	and/or	Secondary Stop Code
Face-to-Face Visit	170, 172, 322, 323, 348, 350	AND	000, 185, 186, 187, 188
		OR	NULL
Secure Messaging	170, 172, 322, 323, 348, 350	AND	719
Video Visits	170, 172, 322, 323, 348, 350	AND	179, 189, 444, 445, 690, 692, 693, 694, 695, 696
Telephone visits	103,147,148,182,708	AND	170, 172, 322, 323, 348, 350
	178, 326, 338	AND	NULL

© 2024 Schuttner L et al. *JAMA Network Open.*

eTable 2. Characteristics of High-Risk Veterans That Remained Engaged in VA Primary Care From 2019-2022

	**Pre-pandemic (n=1,383,070)**	**Year 1 (n=1,250,438)**	**Year 2 (n=1,129,683)[a]**
Age (y), median	73	73	72
≥ 85 years	256,041 (18.5)	204,174 (16.3)	156,287 (13.8)
Male	1,278,244 (92.4)	1,149,069 (91.9)	1,030,192 (91.3)
Race and ethnicity[b]			
Asian, non-Hispanic	7,378 (0.6)	6,755 (0.6)	6,204 (0.6)
Black, non-Hispanic	273,084 (20.5)	254,011 (21.0)	236,774 (21.7)
Hispanic	93,612 (7.0)	86,435 (7.2)	79,848 (7.3)
NH/PI/ AN/AI, non-Hispanic	18,156 (1.4)	16,604 (1.4)	15,137 (1.4)
White, non-Hispanic	941,425 (70.6)	843,314 (69.9)	752,005 (69.0)
Married	638,419 (46.2)	571,841 (45.7)	511,271 (45.3)
Chronic conditions, mean (SD)	4.7 (2.2)	4.3 (2.2)	4.4 (2.2)
Serious mental illness	77,181 (5.6)	38,142 (3.1)	44,531 (4.0)
Substance use disorder	194,799 (14.1)	100,456 (8.2)	109,840 (10.0)
High disability[c]	517,276 (37.4)	425,921 (34.1)	495,261 (43.9)
Hospitalized in prior year[d]	39,713 (2.9)	58,614 (4.8)	54,037 (4.8)
Homelessness	40,235 (2.9)	19,608 (1.6)	12,142 (1.1)
Drive distance to PC in miles, mean (SD)	15.2 (14.8)	15.1 (14.7)	15.0 (14.6)
Geography			
Urban	921,098 (66.6)	835,357 (66.8)	755,521 (66.9)
Rural	405,682 (29.3)	365,746 (29.3)	329,489 (29.2)
Highly rural/Islands[e]	56,290 (4.1)	49,335 (4.0)	44,673 (4.1)
CBOC	725,173 (52.5)	654,212 (53.2)	594,391 (53.3)
Internet speed			
Optimal	515,614 (37.7)	471,473 (37.8)	427,247 (37.8)
Adequate	752,270 (55.0)	685,223 (54.9)	617,947 (54.7)
Inadequate[e]	115,186 (8.3)	93,742 (7.5)	84,489 (7.5)

[a]584 patients missing Year 2 descriptive data, among 1,129,683. [b]Other/unknown not shown, includes multiple or response declined. [c]VA priority group is a military service-related disability or income determination. [d]All-cause, acute hospitalizations (≥ 1) in 12 months prior. [e]Includes unknown (≤ 1%). CBOC = Community-based outpatient clinic. NH/PI/AN/AI = Native Hawaiian, Pacific Islander, Alaska Native, American Indian. PC = primary care.

© 2024 Schuttner L et al. *JAMA Network Open.*

eFigure. Telehealth Use Among High-Risk Veterans Engaged in Primary Care Throughout 2019-2022, Adjusted Relative Risk Ratios (95% CI) Compared to Never Users (All Modelled Results)

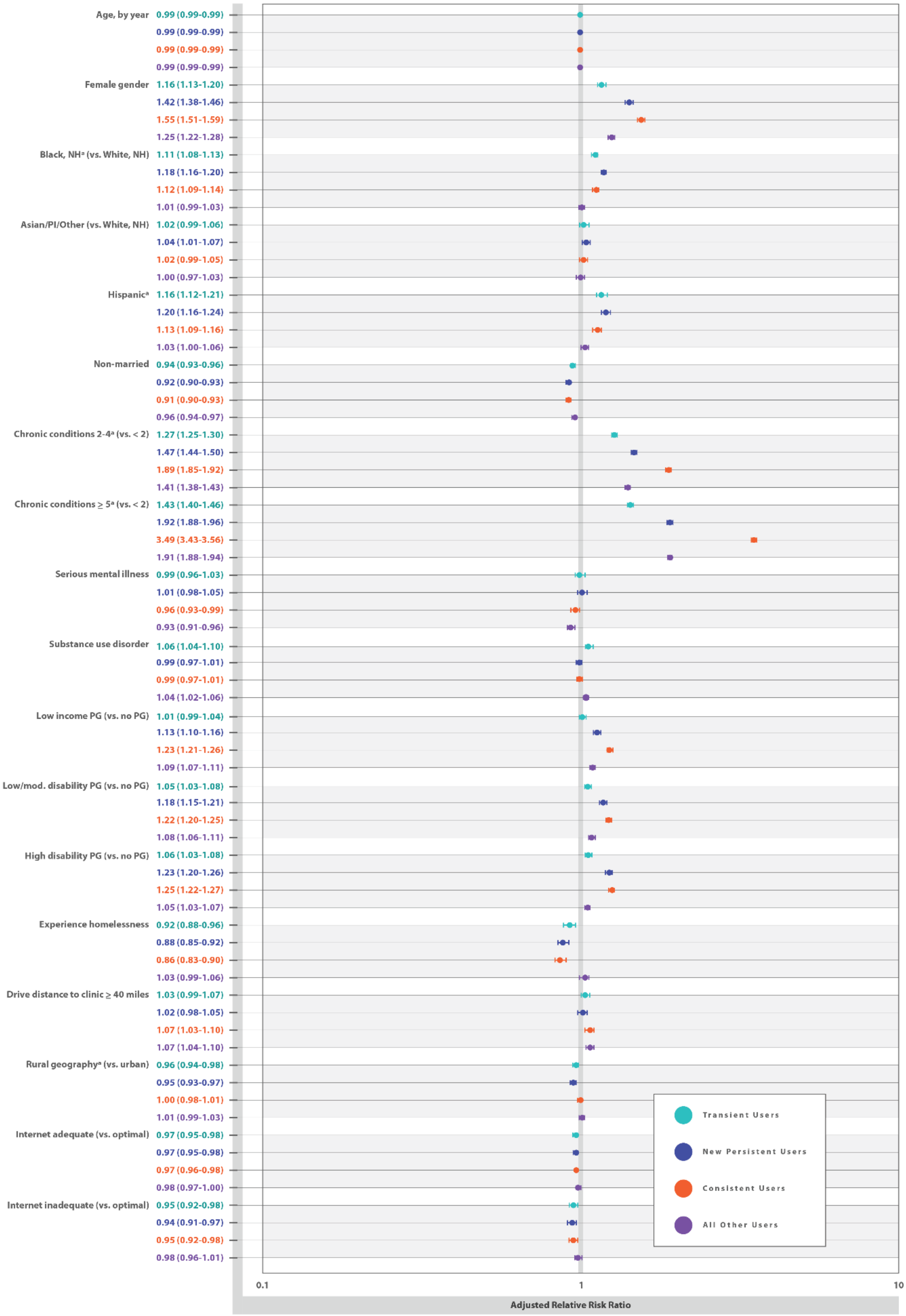

eFigure 1 footnote. NH = Non-Hispanic. PG = VA Priority Group. Patients missing continuous covariates were excluded from models (n=2,201, 0.2%). Models also adjusted for primary care staffing ratio and community vs. hospital affiliation. [a]Combined levels due to small cell sizes.

© 2024 Schuttner L et al. *JAMA Network Open.*
