# Supplementary material for: Primary Care Telehealth Initiation and Engagement Among Veterans at High Risk, 2019-2022
Source: JAMA Netw Open. 2024 Jul 31;7(7):e2424921. doi: 10.1001/jamanetworkopen.2024.24921 (PMC11292453; doi:10.1001/jamanetworkopen.2024.24921)
Supplement: Supplement 2. — Data Sharing Statement [file jamanetwopen-e2424921-s002.pdf]

Data Sharing Statement

Schuttner. Primary Care Telehealth Initiation and Engagement Among Veterans at High Risk, 2019-2022. *JAMA Netw Open*. Published July 31, 2024.
doi:10.1001/jamanetworkopen.2024.24921

Data
Data available: No

Additional Information
Explanation for why data not available: Data for this study was collected as part of a national evaluation effort through the Veterans Health Administration Office of Primary Care. As such, no datasets will be available for dissemination or sharing outside the institution. Analytic plans and detailed methods, outside those shared through scientific publications and conference proceedings, can be made available upon reasonable request.
